# Tumour budding is a novel marker in breast cancer: the clinical application and future prospects

**DOI:** 10.1080/07853890.2022.2070272

**Published:** 2022-05-10

**Authors:** Ting Huang, Hui Bao, Yu-hua Meng, Jian-lin Zhu, Xiao-dong Chu, Xiao-li Chu, Jing-hua Pan

**Affiliations:** a Department of Clinical Pathology, the First Affiliated Hospital of Jinan University, Guangzhou, China; b Department of Plastic Surgery, the First Affiliated Hospital of Jinan University, Guangzhou, China; c Department of Clinical Pathology, the First People's Hospital of Shunde, Foshan, China; d Department of General Surgery, the First Affiliated Hospital of Jinan University, Guangzhou, China; e State Key Laboratory of Dampness Syndrome of Chinese Medicine, The Second Affiliated Hospital of Guangzhou University of Chinese Medicine, Guangdong, China; f Guangdong Provincial Key laboratory of Chinese Medicine for Prevention and Treatment of Refractory Chronic Diseases, Guangzhou, China

**Keywords:** Breast cancer, epithelial–mesenchymal transition, tumour budding

## Abstract

Breast cancer (BC) is a group of markedly heterogeneous tumours. There are many subtypes with different biological behaviours and clinicopathological characteristics, leading to significantly different prognosis. Despite significant advances in the treatment of BC, early metastatic is a critical factor for poor prognosis in BC patients. Tumour budding (TB) is considered as the first step process of tumour metastasis and is related to the epithelial–mesenchymal transition (EMT). TB has been observed in a variety of cancers, such as colorectal and gastric cancer, and had been considered as a distinct clinicopathological characteristics for early metastasis. However, TB evaluation standards and clinical application are not uniform in BC, as well as its molecular mechanism is not fully understood. Here, we reviewed the interpretation criteria, mechanism, clinicopathological characteristics and clinical application prospects of TB in BC.

**Key messages**
- Currently, tumour budding is a poor prognosis for various solid tumours, also in breast cancer.
- Tumour budding is based on epithelial-mesenchymal transition and tumour microenvironment factors and is presumed to be an early step in the metastatic process.
- Breast cancer tumour budding still needs multi-centre experiments. We summarize the current research on breast cancer tumour budding, analyse the method of discriminating breast cancer tumour budding and explore the prognostic role and mechanism in breast cancer.

## 1. Introduction

Breast cancer (BC) is one of the most common cancers in women [1], with high heterogeneity in its morphology, molecular expression profile and clinical course. Doctors choose endocrine or targeted therapy according to the molecular expression of the oestrogen receptor (ER), progesterone receptor (PR) and human epidermal growth factor receptor 2 (HER-2), which greatly improve the overall survival of BC patients. However, invasion and early metastasis are critical hallmarks for poor prognosis in BC [2]. As previous study report, approximately 30% of BC patients had distant metastases in the first diagnosis. Moreover, current pathological characteristics cannot fully reflect the early metastases biological behaviour in BC, such as the tumour differentiation, vascular infiltration, TNM staging. Therefore, alternative or additional histopathological features are required to predict early metastasis and prognosis of BC patients.

Tumour budding (TB) is usually defined as isolated single cancer cells or clusters of up to four cancer cells located at the invasive tumour front [3]. TB provides a histological basis for tumour invasion and metastasis [4]. TB is an emerging prognostic biomarker in colorectal cancer (CRC) and other solid cancers, including BC. Multiple studies have shown that TB is a prognostic marker of early-stage cancer [5,6]. Recently, studies have shown that TB is associated with poor clinicopathological characteristics, such as tumour size, tumour differentiation, lymph node invasion, lymphatic or vascular invasion [7–12]. However, the molecular mechanism and clinicopathological characteristics of TB in breast cancer are still unclear. Therefore, this article will review TB evaluation methods, molecular mechanisms and prospects in clinical applications of BC.

## 2. Tumour budding: Concept and methodologies

**Figure 1.**
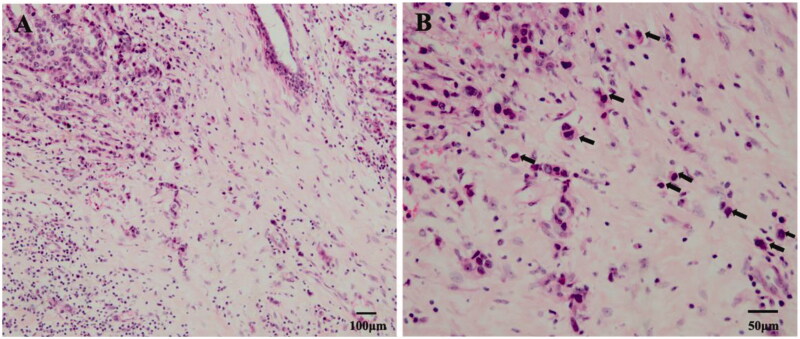
Tumour budding with haematoxylin and eosin (H&E) staining in breast cancer. (a) In the tumour infiltration frontier, we can see a single isolated cancer cell or a cancer nest composed of less than five cancer cells (100×). (b) Tumour buds were observed at 200× using the International Tumour Budding Consensus Conference (ITBCC) recommended method, as shown by the black arrow (200×).

TB is a pathological phenomenon, defined as a single isolated cancer cell or a cancer nest composed of less than five cancer cells located mainly (but not completely) in the aggressive front edge of the tumour stroma [3] (Figure 1). In 1949, a Japanese researcher, Imai, first described this phenomenon in gastric cancer. Pathologists can observe TB by haematoxylin and eosin (H&E) staining, immunohistochemical (IHC) staining such as pan-cytokeratin (Pan-CK) can also be used to identify epithelial cells in poorly differentiated or severely necrotic tumours.

The International Tumour Budding Consensus Conference (ITBCC) recommends the use of counting methods to evaluate TB in CRC. The different TB grades can be distinguished by selecting hot spots for counting (×20 objective lenses, 0.785 mm^2^ fields of view area), and dividing the tumour buds into three levels: 0–4 is low grade (BD1), 5–9 is medium grade (BD2) and ≥10 is high grade (BD3) [9]. Moreover, the quantification criteria for TB in other types of solid cancer specimens, such as biopsy specimens and surgical resection specimens, are still inconsistent with the criteria.

There is also no quantification criteria of TB grading classification in BC (Summary in Table 1). Renuka et al. [13] used the ITBCC in 2016 to evaluate the TB grading in BC. Tumour buds were counted on one hotspot (a field measuring 0.785 mm2) at the invasive front, and high-grade TB was classified as >4 tumour buds per 0.785 mm^2^ hotspot [3]. Three studies [14–16] used the average tumour buds count across 10 high-power fields (HPF). The subsequent thresholds set for high-grade TB were Gabal et al. [14] and Salhia et al. [15] ≥ 4 tumour buds and Masilamani et al. [16] ≥ 10 tumour buds. Liang et al. [17] used receiver operating characteristic (ROC) analysis to determine that seven tumour buds per 0.950 mm2 field size was indicative of high-grade TB. Gujam et al. [18] used Kaplan-Meier analysis to set a cutoff value of >20 tumour buds per 5 HPFs for high-grade TB. Agarwal et al. [19] screened for the area of the highest density of tumour buds, and the threshold for high-grade TB was set at ≥10 tumour buds. So, TB grading is no standard classification in BC, which needs future multicentre retrospective clinical studies and prospective randomized clinical trials.

**Table 1.** Relationship between tumour budding and clinicopathological characteristics in breast cancer.

**General information**	Liang 2013	Gujam 2015	Salnia 2015	Gabal 2018	Agarwal 2019	Masilamani 2019	Renuka 2019
Study type	Restrospective	Restrospective	Restrospective	Cross-seltional observational	Prospective	Restrospective	Prospective
Study origin	China	Swtland	Switzerland	Egypt	India	India	India
High-grade TB evaluation	≥7 tumour buds per 0.950mm^2^ field	>20 tumour buds per 5 HPF	Mean tumour buds >4 in 10 HPF	Mean tumour buds ≥4 in 10 HPF	≥10 tumour buds in area of highest TB densty	Mean tumour buds ≥10 in 10 HPF	>4 tumour buds per 0.785mm^2^ field
Tumour type	IDC-NOS	IDC	IBC-NST	IDC	Breast carcinoma	Breast carcinoma	IBC-NST
*N*	160	474	148	61	40	107	50
**Clinicopatholo-gical characteristics**							
Tumour size	+	–	NR	–	+	NR	–
Tumour grade	–	–	NR	–	–	–	–
LNM	+	+	+	+	–	+	+
LVI	+	+	+	–	NR	+	+
BVI	NR	–	NR	NR	+	NR	NR
ER	NR	+	NR	–	–	–	–
PR	NR	–	NR	–	–	–	NR
HER-2	–	–	NR	–	–	+	NR
Molecular subtypes (LuminalA/Lu-minalB/ HER-2+/TNBC)	NR	–	NR	NR	NR	–	NR
**Survival analysis**							
Multivariate analysis	HR (95%CI)=4.275 (2.99-7.949) *p*<.001	HR (95%CI)=1.96 (1.14-3.09) *p*<.004	NR	NR	NR	NR	NR

*N*: number of patients; IDC-NOS: invasive ductal carcinoma not otherwise specified (NOS); IBC-NST: invasive breast cancer no specific type; LNM: lymph node metastasis; LVI: lymph vessel invasion; BVI: blood vessel invasion; ER: oestrogen receptor; PR: progesterone receptor; HER-2: human epidermal growth factor receptor 2; TNBC: triple-negative breast cancer; HPF: high-powered field; HR: hazard ratio; CI: confidence interval, correlation between TB and clinical characteristics, "+"*p* < .05, "−"*p* > .05, *NR*: not reported.

Due to the inconsistent quantification standards of TB in BC, different studies show different clinicopathological characteristics and prognosis. In Table 1, all seven of the included studies proved the association between TB and lymph node metastasis (LNM), lymphovascular invasion (LVI). Significant correlation between high-grade TB and LNM was reported in six of the seven included studies [13–18]. Six studies reported a significant increased risk of LVI in presence of high-grade TB [13,15–19]. Histological grade of BC was not associated with high-grade TB in six studies [13,14,16–19]. Only two studies have shown a correlation between tumour size and high-grade TB [17,19]. For hormonal status and molecular subtypes, only one study showed high-grade TB association with ER [18], HER-2 [16], whereas other studies showed no correlation. The development of international, evidence-based, standardized scoring systems for TB is essential for future multicentre retrospective clinical studies and prospective randomized clinical trials to better define the different prognostic groups [20].

Evaluation of TB can easily provide useful feedback on the patient’s clinical situation, which can then be easily disseminated from pathologist to clinical physician, because it can be examined in the H&E-stained specimens as a part of conventional pathologic diagnosis. At present, there are the following problems in the interpretation of buds in BC. (1) Are the cell clusters <5 observed in a single paraffin section truly budding? Studies of 3D reconstruction models of CRC, pancreatic cancer(PDAC), lung cancer and BC have shown that many tumour bud clusters are still related to tumour blocks, and that there are few "true" isolated tumour buds (9–22%) [21]. Therefore, the single-cell invasion may be a very rare event, and serial paraffin sections are recommended. After continuous paraffin sections of BC, single cell or less than five cluster cells were observed in the same field of view as true tumour buds. (2) The breast is rich in fibrous and fatty tissue and has no clear histological hierarchical structure. Therefore, it is difficult to judge the "invasive front edge" especially in biopsy specimens. (3) BC has obvious interstitial reactions, such as inflammatory cell infiltration, fibroblast proliferation, tumour necrosis, etc., all of which increase the difficulty of interpreting TB. When inflammatory cells affect the recognition of buds or bud cells, morphology changes from round to spindle type through the epithelial–mesenchymal transition (EMT) process, which may be very similar to the morphology of myofibroblasts. In the presence of neoplastic necrosis, cell debris increases and the interpretation of the neoplastic necrotic area may cause erroneous. (4) TB also needs to be differentiated from poorly differentiated cell clusters in H&E staining section. Poorly differentiated cells clusters are often nest-shaped and have more than five cells.

## 3. The mechanism of TB formation

### 3.1. EMT of cancer

**Figure 2.**
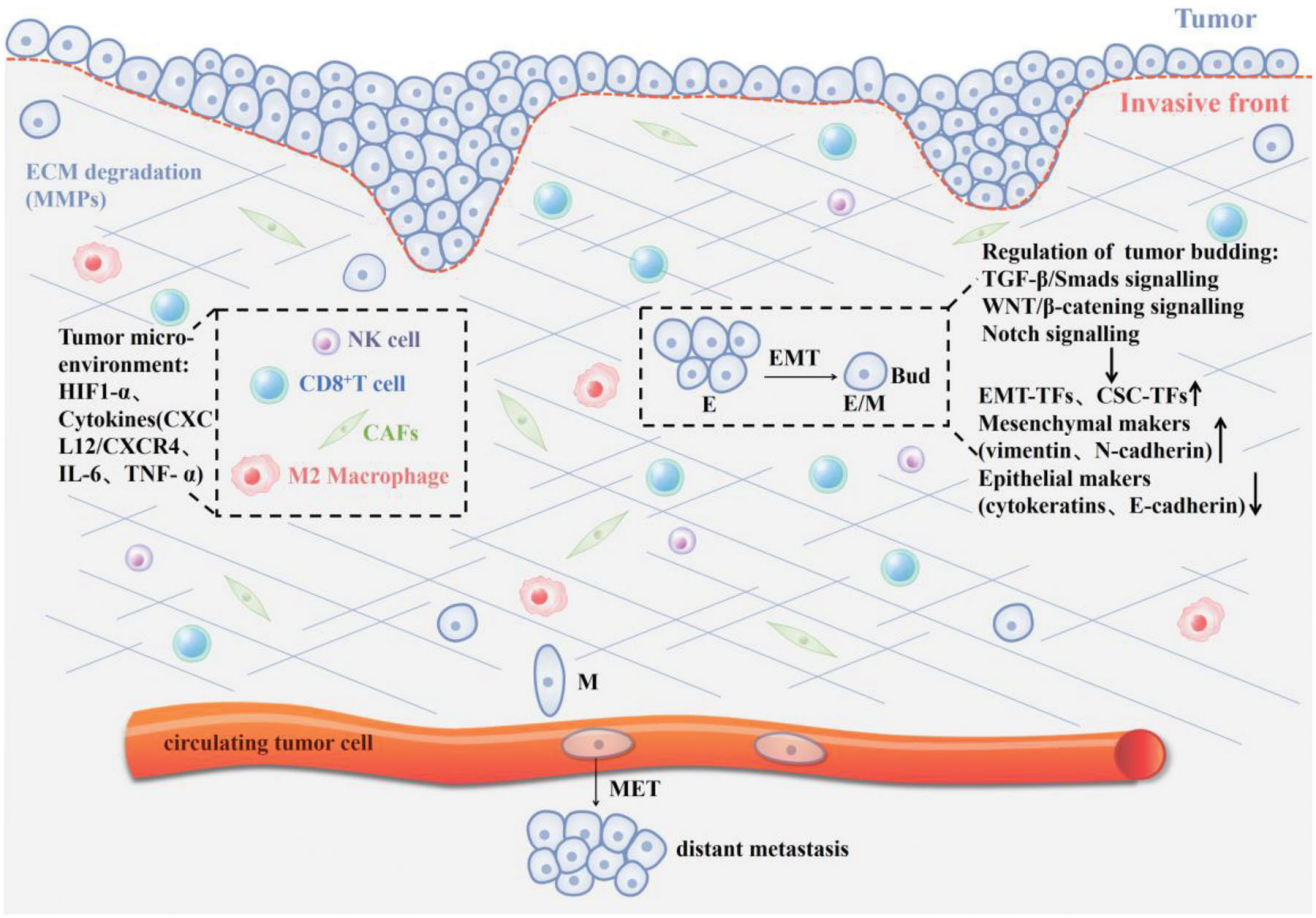
The mechanism of tumour budding: 1. Regulate the phenotype of tumour cells through TGF- β/SMADS, Notch, WNT/β-catenin and other signal pathways, thereby inducing the EMT process, such as epithelial marker CK downregulation, mesenchymal marker vimentin upregulation, EMT-related transcription factors (ZEB, Twist, Snail) and cancer stem cell markers were up-regulated, and cell adhesion loss (E-cadherin down-regulated); 2.Hypoxia and various cytokines in the tumour microenvironment affect tumour budding.

Activating invasion and metastasis are hallmarks of cancer [2]. TB is strongly associated with EMT and various factors in the tumour microenvironment, where individual tumour buds interact with diverse components of the tumour stroma and immune system. The EMT is associated with cancer invasion and progression [22]. EMT is considered as a key driving factor for embryonic development, tissue fibrosis, wound healing, tumourigenesis and metastasis [23]. EMT types: Type I usually occurs during embryonic development; Type II is related to wound healing, tissue regeneration and organ fibrosis; Type III occupies an important position in epithelial cancer cells and participates in metastasis and cancer progression [24]. EMT is activated in a variety of malignant tumour cells, leading to the loss of typical epithelial (Epi) cell characteristics (cell–cell connection and apical-basal polarity) and the obtaining of mesenchymal (Mes) cell characteristics. EMT is described as a dynamic process, and often described as partial or “hybrid” EMT, whereby the loss of epithelial characteristics and acquisition of mesenchymal traits is temporary, rather than permanent. Finally, EMT process enables tumour cells to become locally invasive, infiltrate lymphatic and/or blood vessels, through the vasculature into the parenchyma, and finally, seed distant micro-metastases [23,25]. There are a variety of signal transduction pathways that are the main regulators of EMT and related to the activation of EMT transcription factors (EMT-TFs), such as SNAIL, ZEB and TWIST [26]. These core EMT-TFs are mechanically activated by TGF-β-SMADS, WNT/β-Catenin signalling, epidermal growth factor (EGF)/fibroblast growth factor (FGF)–receptor tyrosine kinase (RTK) signalling, Notch signalling and the MAPK pathway, further initiating EMT-associated changes in gene expression [27]. The following pathways are briefly described (Figure 2).

### 3.2. TGF-β signalling pathway

The TGF-β signalling pathway plays an important role in the invasion and metastasis of BC. TGF-β is a relatively simple membrane receptor with a nuclear transcriptional activation pathway that plays a crucial role in many biological events, such as the self-renewal and differentiation of embryonic stem cells, the homeostasis of differentiated cells, the suppression of the immune system and the development of cancer [28]. In normal and premalignant cancer cells, the TGF-β signalling pathway inhibits proliferation and promotes cell cycle arrest and apoptosis. However, the activation of this pathway in advanced cancer cells can promote the transformation of epithelial cells to mesenchymal cells, regulate cell stemness and activity and enhance tumourigenesis and metastasis [29]. The extracellular TGF-β signals via heteromeric complex of transmembrane TGF-β receptors 1 and 2 (TβR1 and TβR2). Upon TβR1 activation, regulatory SMADs (R-SMADs) become phosphorylated and form heteromeric complexes with SMAD4. R-SMAD/SMAD4 complexes can act as transcription factors in concert with coactivators [30].

Downstream of TβR signal transduction, the phosphorylation of SMADs activates the transcription of ZEB, TWIST and SNAIL family members, thereby inducing transcriptional repression of E-cadherin [20]. Non-canonical EMT TGF-β signalling is represented by several pathways, including NF-κB, Par6, small GTPases, PI3K/AKT/TOR and MAPK family with activation of ZEB-1. These pathways regulate distinct processes including cytoskeleton organization, cell survival, migration and invasion [31,32]. Also, MMP-9 is a downstream effector of TβR1. Inhibition of MMP-9 can prevent TβR1-induced EMT and reduce cell invasion and metastasis [33]. TGF-β overexpression in TB of CRC and dysregulation of SMAD has been demonstrated [34].

### 3.3. Notch signalling pathway

A study found that knocking out Slug in the BC cell line reduced vimentin expression and increased E-cadherin expression, thereby reversing the EMT process and reducing the invasive ability of BC cells [35,36]. Recent studies have shown that activation of Notch/PI3K/Akt/NF-κβ axis up-regulates NF-κβ transcriptional activity and increases TB in BC by promoting EMT [33,37].

The Notch signalling pathway is an original signal path regulating the differentiation and development of cells, tissues and organs. Any abnormality in the Notch signalling pathway will inevitably lead to fatal results, such as cancer. Notch regulates the occurrence and development of breast cancer. One of the key processes is EMT. Activation of the Notch signalling pathway is mainly manifested by downregulation of endothelial markers Tie1, Tie2, platelet-endothelial cell adhesion molecule-1 and endothelial NO synthase, as well as mesenchymal marker overexpression such as α-SMA, fibronectin and platelet-derived growth factor receptor (PDGFR). In conclusion, several studies have shown that the activation of Notch signal can promote EMT to further induce TB in BC.

### 3.4. Wnt/β-catenin signalling pathway

Suppression of E-cadherin expression has been studied in TB, including the ZEB1, ZEB2, TWIST1, TWIST2, SNAI1 (SNAIL) and SNAI2 (SLUG) [38–40]. In invasive non-specific ductal carcinoma (IDC-NST), E-cadherin membrane localization expression of tumour buds was low [17].

The WNT signalling pathway plays an important role in the axial differentiation of multicellular organisms. It is composed of the WNT ligand, WNT receptor, β-catenin, glycogen synthase kinase 3 (GSK-3), APC protein, etc. The classic WNT/β-catenin pathway plays an important role in the WNT pathway, which activates target genes' transcriptional activity through the nuclear translocation of β-catenin. Under normal circumstances, the WNT pathway is closed. When the WNT pathway is activated, cells proliferate and differentiate abnormally, leading to tumour formation. β-catenin participates in the WNT signalling pathway and forms a complex with E-cadherin and localizes on the cell membrane to maintain cell adhesion. Activation of the WNT/β-catenin pathway can induce SNAIL expression, then downregulate E-cadherin and upregulate the expression of vimentin, further inducing the EMT process [41].

### 3.5. Others (tumour microenvironment, hypoxia, microRNA, cell stemness)

TB is strongly associated with EMT and various factors in the tumour microenvironment (TME), where individual tumour buds interact with diverse components of the tumour stroma and immune system. TME is characterized by hypoxia, acidity, inflammation and immunosuppression [42,43]. In addition, the established tumours often exist with an immunosuppressive microenvironment that can block productive antitumour immunity [44]. The immune cells in TME secrete cytokines, inflammatory factors and chemokines through various channels to drive cancer's EMT process. In turn, cancer cells can also interact with immune cells to induce cell plasticity, release immunosuppressive substances, create an immunosuppressive microenvironment and promote invasion and metastasis [42]. The interaction between tumour buds as tumour-related factors and immune cells as host-related factors reflects the aggressor-defender model and has important prognostic and potential therapeutic implications. Furthermore, EMT status has been confirmed to be related to the activation of different immune checkpoint molecules, and the EMT score may provide a new predictive biomarker for the clinical response of immune checkpoint blockade [45,46].

Hypoxia is the main physiological driving factor of EMT. The main changes include the upregulation of hypoxia-inducible factor 1-α (HIF1-α), hepatocyte growth factor (HGF), SNAI1 and TWIST1. Moreover, increased expression of hypoxia-related genes is associated with poor prognosis. The hypoxic microenvironment plays a key role in regulating BC progression and metastasis [47].

During the progression of BC, miRNAs play a role in regulating gene expression. Compared with normal tissues, the levels of most miRNAs in cancer tissues are decreased. miRNA-200c has been shown to inhibit EMT by targeting ZEB1/ZEB2 and maintaining E-cadherin expression [48,49]. Rhodes *et al.* have shown that Mir-200 family members can directly target and inhibit CDH1, ZEB1 and ZEB2 to induce EMT [48].

The survival of cancer cells depends on the mechanism against cell death, especially apoptosis. Tumour cells can resist apoptosis and have the ability to self-renew. Tumour buds usually overexpress stem cell markers, such as LGR5, ALDH1, and CD44, which suggests that they may have the ability to self-renew no matter whether in the primary site or distant metastatic tissues [50–52]. However, there is heterogeneity in the invasion and metastasis potential of tumour bud cells.

## 4. The application prospect of TB in BC

The 2016 ITBCC issued grading recommendations to standardize pathologists' assessment and reporting of CRC buds. First, moderate or high tumour budding is an independent predictor of lymph node metastasis in the pT1 CRC and is increasingly considered (along with other clinicopathological factors) when deciding whether radical surgery is required, rather than local resection of the tumour. Second, in patients with stage II colon cancer, a high tumour budding rate is a strong poor prognostic factor (high-risk feature), and adjuvant chemotherapy should be considered [3]. At the same time, the consensus recommends a 3-level stratification system. Both BD2 and BD3 are risk factors for lymph node metastasis in patients with pT1 (stage I) CRC, whereas only BD3 is associated with an increased risk of recurrence and death in patients with stage II CRC [3]. Therefore, the tertiary stratification system better reflects the continuity of TB. The latest study of 771 CRC patients showed that the level of TB has nothing to do with tumour grade. And in terms of predicting disease-free survival, TB was better than tumour grade [53]. However, the effect of tumour budding on the clinicopathological characteristics of patients in BC requires larger-scale and multi-centre research.

In 474 patients with invasive ductal carcinoma of the breast, TB was associated with poor clinicopathological features, including larger tumour size, LVI, lymphovascular invasion, LNM, high tumour stroma content, low inflammatory infiltration, and higher buds were also associated with lower five-year overall survival (OS) and shorter cancer-specific survival (CSS) [17,18]. Budding is significantly correlated with known adverse histological features (mainly LVI) and lymph node positivity [54]. Therefore, in the total specimen sections, the level of TB can be used to predict lymphatic invasion and lymph node involvement. Salhia et al. investigated intratumoral (ITB) and peripheral tumour buds (PTB) can be used as predictors of lymph node involvement by comparing 148 resected specimens of invasive ductal carcinomas with 99 matched preoperative biopsy specimens [15]. Thus, it is possible to predict further whether patients' lymph nodes are involved and the subsequent surgical approach is based on their ITB biopsy level.

The main tumour and tumour buds may have co-driver mutations [55]. Laedrach *et al.* demonstrated that BC budding cells could have the same ER and HER-2 expression profiles as those of the main tumour [56], indicating that the tumour buds and the main tumour may have the same response to clinical treatment. In addition, TB may be driven by hormones, leading to different types of BC tumours with different budding levels. The latest meta-analysis study showed that ER and HER-2 positive tumours have higher levels of TB, whereas triple-negative breast cancers (TNBC) have lower levels [54]. Although many key EMT proteins upregulated TNBC, no correlation between TNBC and high-grade budding was found [57]. This indicates that there are other alternative metastasis processes in this more aggressive phenotype. At the same time, the high expression of transcription factors during TB EMT may increase chemotherapy resistance [58]. Therefore, can we predict chemotherapy resistance based on the expression level of transcription factors in tumour buds? Studies have shown that circulating tumour cells (CTCs) are enriched in BC cells with EMT characteristics and are associated with increased risk of recurrence and decreased OS after chemotherapy [59]. CTCs are a manifestation of TB, so we can predict CTCs based on TB to further analyse the recurrence and metastasis risk in BC patients.

Regardless of the clinical scenario or tumour type, the assertion that "the more tumour buds, the worse the clinical outcome" applies. TB is presumed to be an early step in the metastatic process. Single or collective cell migration caused carcinoma *in situ* to invasive carcinoma in several different tumour types, in which TB with mesenchymal phenotype takes outstanding position.

## 5. Perspectives

TB can predict lymphatic invasion and lymph node involvement and play an increasingly prominent role in recurrence, metastasis and chemotherapy resistance. Therefore, more research is needed to explore the role of TB in BC. In the future, we should strengthen the exploration of the molecular and pathogenic mechanism of TB and conduct multifactor analysis and clinical trials on this basis to clarify its value, further provide the basis for the judgement of BC recurrence and clinical prognosis and develop "anti-budding therapy". By understanding EMT's mechanism, we can better explore the potential progression from ductal carcinoma *in situ* to invasive BC and finally to metastasis. At the same time, the presence of sentinel lymph node metastasis can be predicted according to the number of TB in different BC molecular types, which further guides the scope of intraoperative lymph node dissection. In addition, we can observe the level of TB to predict the clinicopathological characteristics of breast cancer, especially whether further chemotherapy was needed in early breast cancer. Thence, TB has broad clinical applications.

## 6. Conclusions

The invasive biological behaviour of tumour buds increases the ability of tumour cells to migrate and invade. TB plays an important role in various cancers. However, TB has not yet entered routine clinical evaluation of BC. There is no consensus on the precise definition of TB in BC, the evaluation method, the best way to stratify and assign patients into prognostic categories and how to make appropriate treatment decisions. Therefore, it is necessary to establish consistent pathological criteria to identify and quantify TB to improve TB counting's accuracy and repeatability in clinicopathological practice. TB is significantly related to lymph node involvement and lymphatic invasion, which can predict patients' prognosis. We need to fully understand the mechanism of TB in BC and further guide and enrich patient treatment options.

## Data Availability

Data sharing is not applicable to this article as no new data were created or analysed in this study.
